# Heterologous Expression of the Nybomycin Gene Cluster from the Marine Strain *Streptomyces albus* subsp. *chlorinus* NRRL B-24108

**DOI:** 10.3390/md16110435

**Published:** 2018-11-04

**Authors:** Marta Rodríguez Estévez, Maksym Myronovskyi, Nils Gummerlich, Suvd Nadmid, Andriy Luzhetskyy

**Affiliations:** 1 Pharmazeutische Biotechnologie, Universität des Saarlandes, 66123 Saarbrücken, Germany; marta.rodriguezestevez@uni-saarland.de (M.R.E.); m.myronovskyi@googlemail.com (M.M.); nils.gummerlich@uni-saarland.de (N.G.); suvdn@yahoo.com (S.N.); 2 Helmholtz-Institut für Pharmazeutische Forschung Saarland, 66123 Saarbrücken, Germany

**Keywords:** streptomycetes, secondary metabolites, nybomycin gene cluster, heterologous expression, nybomycin biosynthesis

## Abstract

Streptomycetes represent an important reservoir of active secondary metabolites with potential applications in the pharmaceutical industry. The gene clusters responsible for their production are often cryptic under laboratory growth conditions. Characterization of these clusters is therefore essential for the discovery of new microbial pharmaceutical drugs. Here, we report the identification of the previously uncharacterized nybomycin gene cluster from the marine actinomycete *Streptomyces albus* subsp. *chlorinus* through its heterologous expression. Nybomycin has previously been reported to act against quinolone-resistant *Staphylococcus aureus* strains harboring a mutated *gyrA* gene but not against those with intact *gyrA*. The nybomycin-resistant mutants generated from quinolone-resistant mutants have been reported to be caused by a back-mutation in the *gyrA* gene that restores susceptibility to quinolones. On the basis of gene function assignment from bioinformatics analysis, we suggest a model for nybomycin biosynthesis.

## 1. Introduction

Actinobacteria represent a prominent source of natural products with potential industrial applications. The genus *Streptomyces* is especially well known to produce a diverse spectrum of compounds with antibacterial, antifungal, antitumor and even insecticide and herbicide activity [1,2,3]. The increasing amount of sequenced microbial genomes has provided insight into the unprecedented potential of actinobacteria to biosynthesize natural products [4,5]. Generally, dozens of various secondary metabolite clusters are encoded in their genomes. However, these clusters are often poorly expressed under standard cultivation conditions or even remain silent, thus preventing the isolation and characterization of the encoded compounds. Such uncharacterized clusters with unknown biosynthetic products are usually regarded as cryptic. Different approaches can be used to characterize cryptic clusters, including changing cultivation parameters (OSMAC approach), expression of pleiotropic regulatory genes, introduction of antibiotic-resistant mutations, and refactoring of the biosynthetic pathways [6,7,8,9]. Currently, characterization of the cryptic gene clusters encoding natural products often relies on expression of their biosynthetic pathways in the optimized surrogate strains called heterologous hosts or chassis strains. The heterologous expression approach has a number of advantages compared to other cluster characterization methods. The simplified metabolic background of the chassis strains facilitates the identification of natural products; fast DNA-recombineering methods in *E. coli* and DNA transfer methods into streptomycetes simplify biosynthetic studies, and high production yields enable product supply for structure elucidation and biological activity studies.

In this study, we report the identification and characterization of the previously uncharacterized nybomycin gene cluster from the marine strain *Streptomyces albus* subsp. *chlorinus* NRRL B-24108 through heterologous expression in *Streptomyces albus* Del14. Nybomycin was first isolated in 1955; however, the unique biological activity of the antibiotic was discovered only recently [10]. Nybomycin inhibits growth of quinolone-resistant *Staphylococcus aureus* by targeting the mutated enzyme gyrase. Interestingly, the intact gyrase encoded by the *gyrA* gene without the resistance mutation is not inhibited by the antibiotic. The rare nybomycin-resistant mutants derived from quinolone-resistant *S. aureus* have all been reported to contain the reverse mutation in the *gyrA* gene, causing loss of quinolone resistance. Despite this interesting mode of action, the biosynthetic gene cluster leading to nybomycin production remains unknown. Based on the cluster analysis, we also propose the biosynthetic route leading to the production of nybomycin.

## 2. Results

### Identification of the Nybomycin Gene Cluster through Its Heterologous Expression

**Figure 1:**
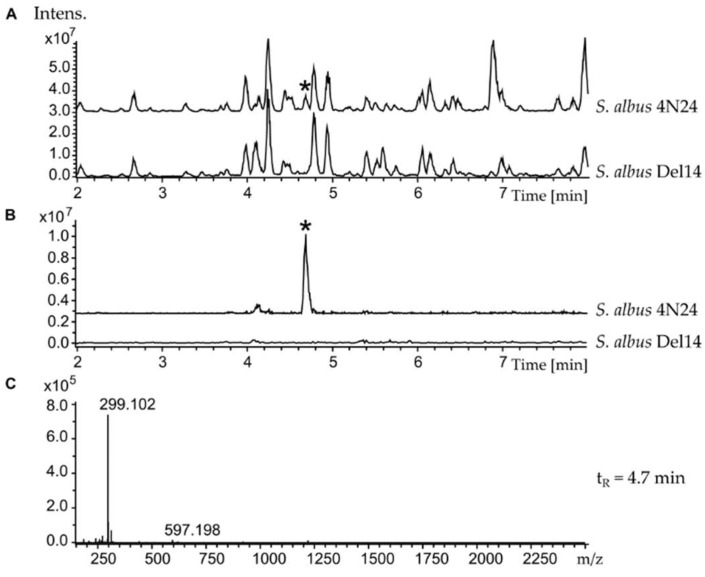
LC-MS chromatograms of crude extracts from *S. albus* 4N24 and *S. albus* Del14. The new peak found in *S. albus* 4N24 crude extract is indicated with an asterisk (*). (**A**) Base peak chromatograms; (**B**) extracted ion chromatograms (299.10 ± 0.1 Da); (**C**) mass spectrum associated to t_R_ = 4.7 min from *S. albus* 4N24 LC-MS chromatogram.

In the course of systematic activation of cryptic secondary metabolite clusters from *Streptomyces albus* subsp. *chlorinus* NRRL B-24108 [3], a cluster annotated by the antiSMASH genome mining software [11] as “fatty acid metabolism cluster” was expressed in the heterologous host strains. For this purpose, a BAC 4N24 containing the cluster was isolated from the previously constructed genomic library of *S. albus* subsp. *chlorinus* and transferred into *Streptomyces albus* Del14 and *Streptomyces lividans* TK24 [12,13]. The obtained exconjugant strains *Streptomyces albus* 4N24 and *Streptomyces lividans* 4N24 as well as the corresponding control strains without the BAC *S. albus* Del14 and *S. lividans* TK24 were fermented in the production medium. LC-MS analysis of the exconjugant strains containing the heterologous cluster confirmed its successful expression in *S. albus* 4N24, as indicated by a new peak that was observed in the extract of the strain (Figure 1A,B and Figure S1). Expression of the cluster in *S. lividans* 4N24 did not lead to the production of any new compounds compared with the control strain.

**Figure 2:**
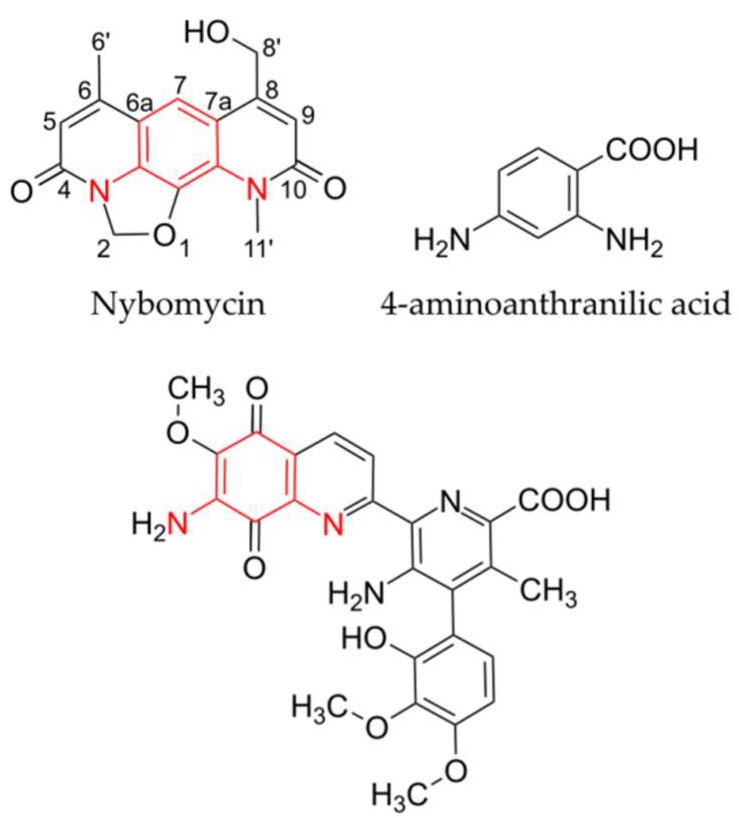
Chemical structures of nybomycin, streptonigrin, and 4-aminoanthranilic acid. The common core structure for nybomycin and streptonigrin are highlighted in red.

Analysis of the *S. albus* 4N24 extract by high-resolution MS analysis revealed that the identified peak corresponded to the compound with an [M + H]^+^ of 299.102 *m*/*z* and the deduced molecular formula C_16_H_15_N_2_O_4_ (Figure 1C). A search in a natural product database revealed that the identified compound might correspond to the antibiotic nybomycin (Figure 2). To verify this, a nybomycin standard (Santa Cruz Biotechnology, Inc., Dallas, TX, USA) was used. LC-MS analysis of the pure nybomycin, the *S. albus* 4N24 extract, and the *S. albus* 4N24 extract spiked with pure nybomycin confirmed that the new compound corresponded to nybomycin; the retention time and the mass spectrum of the new compound were identical to those of the pure standard (Figure S2). Additionally, to validate the identity of the detected compound as nybomycin, the former was purified. For this purpose, *S. albus* 4N24 was inoculated into 10 L of DNPM medium, and the culture broth of the strain was extracted with ethyl acetate. The compound was purified from the extract using normal-phase, size-exclusion, and reverse-phase chromatography. The purified compound, as well as the pure nybomycin standard, was used for NMR measurements. The recorded ^1^H-NMR spectra of the purified compound and of nybomycin were identical (Table S1; Figure S3), which unambiguously proved the identity of the former as nybomycin.

**Figure 3:**
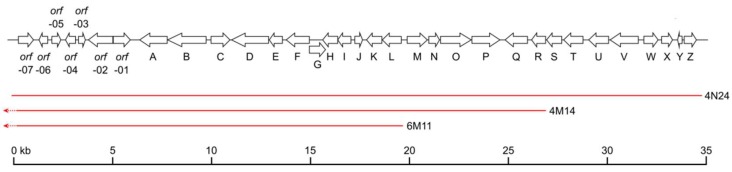
Nybomycin biosynthetic gene cluster. Genetic organization is shown. Below, maps of three different BAC clones are displayed: BAC 4N24 contains the full sequence of the nybomycin gene cluster. BAC 4M14 lacks the genes from *nybS* to *nybZ*; and BAC 6M11 lacks the genes from *nybM* to *nybZ*.

BAC 4N24, which contains the nybomycin biosynthetic genes, comprises a 36 kb genomic DNA region with 33 open reading frames (Figure 3, Table 1). A sequence similarity search revealed that nine open reading frames within this region shared homology at the protein level with the genes involved in the biosynthesis of streptonigrin, which is structurally related to nybomycin (Figure 2). Sequence analysis of the DNA fragment cloned in BAC 4N24 revealed the putative streptomycin-resistant gene, a hypothetical gene, a gene encoding an ATP-binding protein, and four additional hypothetical genes followed by the *nybA* gene encoding a putative 3-carboxy-cis,cis-muconate cycloisomerase at its 5’ end (Table 1), which implied that the *nybA* gene might constitute the 5’ end of the nybomycin cluster. The 3’ end of the cloned region comprised the genes encoding a putative methyltransferase, two isopenicillin N synthases, a transporter, three transcriptional regulators, and a hypothetical protein (*nybS*, *nybT*, *nybU*, *nybV*, *nybW*, *nybX*, *nybZ*, and *nybY*, respectively). To clarify whether these genes were part of the nybomycin biosynthetic cluster, two BACs 4M14 and 6M11 that partially overlap with BAC 4N24 were isolated from the genomic library of *S. albus* subsp. *chlorinus* and expressed in *S. albus* Del14. Both the isolated 4M14 and 6M11 BACs completely covered the 5’ end of the fragment cloned in the original BAC 4N24, which led to nybomycin production. Compared with BAC 4N24, BAC 4M14 lacked the region downstream of the *nybR* gene, while BAC 6M11 lacked the region downstream of the *nybL* gene (Figure 3). The obtained strains *S. albus* 4M14 and *S. albus* 6M11 were analyzed together with *S. albus* 4N24 for nybomycin production. During the LC-MS analysis, no nybomycin was detected in the extracts of either the *S. albus* 4M14 or *S. albus* 6M11 strains (Figure S4). Nybomycin was readily detectable in the extract of *S. albus* 4N24. These results give evidence that the 3’-terminal region of 4N24, which contains the genes downstream of *nybR* (*nybS* to *nybZ*), is essential for nybomycin production. Taken together, our results suggest that the genes from *nybA* to *nybZ* might constitute the nybomycin gene cluster, which is further supported by the fact that the genes from *nybA* to *nybF* and *nybN* to *nybP* share high levels of homology with genes in the biosynthetic cluster of streptonigrin (Table 1), a compound that is structurally similar to nybomycin (Figure 2).

## 3. Discussion

The antibiotic nybomycin was discovered in 1955 in the culture broth of streptomycetes A 717 [14]. The structural features of nybomycin as a fused pyridoquinolone ring system and an angularly fused oxazoline ring are of particular biosynthetic interest as they have not been reported for other natural products [15]. Despite the unique structure of nybomycin, its biosynthetic cluster and biosynthetic route have remained elusive. Only the results of feeding studies imply that acetate, methionine, and some non-identified shikimate-type intermediates serve as biosynthetic precursors for nybomycin [15,16]. In this article, we have described the identification of the nybomycin biosynthetic gene cluster from the marine streptomycete *S. albus* subsp. *chlorinus* NRRL B-24108 through its expression in the cluster-free heterologous host *S. albus* Del14.

Sequence analysis of the DNA fragment cloned in BAC 4N24 has revealed that a number of genes within this fragment are highly homologous to the genes involved in biosynthesis of the antibiotic streptonigrin (Table 1) [17]. Direct comparison of nybomycin and streptonigrin has shown distinct structural similarity, with both structures containing a diamino-substituted, six-membered ring (Figure 2). The structural similarity and the partial homology of the gene clusters suggest that nybomycin and streptonigrin biosynthetic routes can have some similar biosynthetic intermediates and enzymatic reactions.

**Figure 4:**
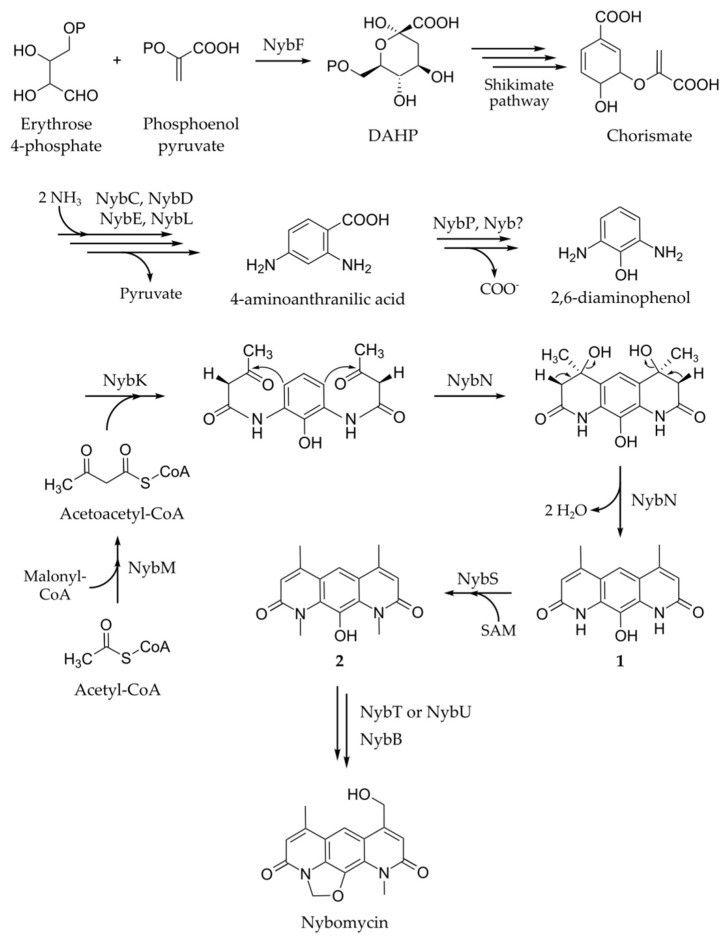
Proposed biosynthetic pathway of nybomycin.

Based on the sequence analysis and the results of BAC expression, we propose that the genes *nybA* to *nybZ* constitute the nybomycin gene cluster. The *nybA* gene, encoding a putative 3-carboxy-cis, cis-muconate cycloisomerase, is homologous to the streptonigrin biosynthetic gene *stnL*. The *nybZ* gene encodes a putative transcriptional regulator that might also participate in nybomycin biosynthesis. Similar to the streptonigrin pathway, the 3-deoxy-d-arabinoheptulosonate 7-phosphate (DAHP) synthase encoded by *nybF* is likely to catalyze the first reaction in the nybomycin biosynthetic route. DAHP synthase is responsible for the first reaction of the shikimate pathway—biosynthesis of 3-deoxy-d-arabino-hept-2-ulosonate 7-phosphate (DAHP) from phosphoenolpyruvate and d-erythrose 4-phosphate (Figure 4) which is supported by the results of feeding experiments that have demonstrated that the carbons of the central ring of nybomycin are derived from a shikimate-type intermediate [15]. Catalyzing the first reaction, DAHP synthase regulates the amount of carbon entering the shikimate pathway and therefore can be responsible for its upregulation to provide sufficient amounts of biosynthetic precursors for nybomycin. The genes encoding enzymes responsible for the conversion of DAHP into chorismate are absent in the nybomycin cluster and in the streptonigrin pathway [17]. Therefore, the host’s primary metabolism enzymes most likely overtake these biosynthetic steps. We propose that the second reaction catalyzed by the enzymes encoded in the biosynthetic cluster is the conversion of chorismate into 4-aminoanthranilic acid (Figure 4), which is a key intermediate in both nybomycin and streptonigrin biosynthesis (Figure 2). Isolation of 4-aminoanthranilic acid from the culture broth of the streptonigrin producer *Streptomyces flocculus* supports this suggestion [18]. Furthermore, 4-aminoanthranilic acid has also been shown to incorporate into streptonigrin [18]. We propose that the products of the *nybC*, *nybD*, *nybE*, and *nybL* genes might be responsible for the conversion of chorismate into 4-aminoanthranilic acid. The *nybL* gene, encoding putative amidohydrolase [19], has no homologue in the streptonigrin biosynthetic pathway. We suggest that the protein product of *nybL* may provide enough supply of ammonia for the amination of chorismate by anthranilate synthase [20], encoded by *nybD*, during the biosynthesis of 4-aminoanthranilic acid. In the streptonigrin biosynthesis, the function of the *nybL* gene may be overtaken by the one of the host’s primary metabolism enzymes.

After formation of 4-aminoanthranilic acid, it is then hydroxylated in the third position and decarboxylated, generating 2,6-diaminophenol (Figure 4). The *nybP* gene encoding putative salicylate hydroxylase might be responsible for the hydroxylation reaction. Then, two acetoacetate units are attached to the amino groups of 2,6-diaminophenol through the action of the *N*-acetyltransferase encoded by the *nybK* gene (Figure 4). The putative acetoacetyl-CoA synthase encoded by the *nybM* gene catalyses the formation of acetoacetyl-CoA from acetyl-CoA and malonyl-CoA. The NybM enzyme is likely responsible for the production of sufficient amounts of acetoacetate, which is used as a precursor in nybomycin biosynthesis. The precursor role of acetoacetate in nybomycin biosynthesis is supported by the results of the feeding experiments, which have unequivocally defined acetate as the source of the exterior carbons of the pyridone rings [16]. We speculate that after attachment of the acetoacetate units, the putative cyclase encoded by the *nybN* gene catalyses the closure of the pyridone rings, leading to formation of intermediate **1** (Figure 4). The methylation of nitrogen within the pyridone rings is likely to be catalyzed by the SAM-dependent methyltransferase encoded by *nybS*. We hypothesize that the next reaction in nybomycin biosynthesis is a closure of the oxazoline ring (Figure 4). This step might be catalyzed by the product of *nybT* or *nybU*, which code for isopenicillin N synthases (IPNS). IPNS is responsible for the biosynthesis of isopenicillin N through a bicyclization reaction—first, the formation of a C-N bond generates a β-lactam ring; then, the closure of a five-membered thiazolidine ring is accomplished by the formation of a C-S bond [21]. Sulfur and oxygen have similar properties [22]. Therefore, in a similar way to thiazolidine ring closure, IPNS could be able to catalyze the formation of a C-O bond between the carbon atom of the *N*-methyl group and the oxygen atom of the OH group present in intermediate **2**, generating an oxazoline ring (Figure 4). Finally, a hydroxylation reaction takes place at C-8’, possibly catalyzed by the oxidoreductase NybB, giving rise to nybomycin final structure. The compound is then secreted to the extracellular space, most likely by the membrane transporter encoded by *nybV*. Expression of secondary metabolite biosynthesis genes is commonly regulated by activators and repressors coded by genes that are located within the same cluster. We hypothesize that the products of *nybW*, *nybX*, and *nybZ*, which encode putative transcriptional regulators, might control the expression of the genes involved in nybomycin biosynthesis.

In this paper we report the identification of the gene cluster encoding production of the structurally unique antibiotic nybomycin. Biological activity of nybomycin is also of particular interest as it inhibits growth of quinolone-resistant *Staphylococcus aureus*, dormant *Mycobacterium tuberculosis*, and other Gram-positive and Gram-negative bacteria [10,14,23]. Interestingly, nybomycin targets solely the mutant, quinolone-resistant DNA gyrase with a Ser84Leu substitution, while it is inactive against the wild-type, quinolone-sensitive form of the enzyme [10]. The mutation described thus far to cause nybomycin resistance is a reverse Leu84Ser mutation within the *gyrA* gene that restores quinolone sensitivity. The identification of the nybomycin cluster presented in this paper enables biosynthetic studies of nybomycin production, generation of new nybomycin derivatives, and optimization of its production as well as a nybomycin supply for further biological studies. Together, these works might help fight the development of quinolone resistance and revive quinolones as an effective class of antibiotics.

## 4. Materials and Methods

### 4.1. General Experimental Procedures

All strains and BACs used in this work are listed in Table S2. *Escherichia coli* strains were cultured in LB medium [24]. *Streptomyces* strains were grown on soya flour mannitol agar (MS agar) [25] for sporulation and conjugation and in liquid tryptic soy broth (TSB; Sigma-Aldrich, St. Louis, MO, USA). For secondary metabolite expression, liquid DNPM medium (40 g/L dextrin, 7.5 g/L soytone, 5 g/L baking yeast, and 21 g/L MOPS, pH 6.8) was used. The antibiotics kanamycin, apramycin and nalidixic acid were supplemented when required.

### 4.2. Isolation and Manipulation of DNA

BAC extraction from a *Streptomyces albus* subsp. *chlorinus* constructed genomic library (Intact Genomics, USA), DNA manipulation, *E. coli* transformation, and *E. coli*/*Streptomyces* intergeneric conjugation were performed according to standard protocols [24,25,26]. Plasmid DNA was purified with the BACMAX™ DNA purification kit (Lucigen, Middleton, WI, USA). Restriction endonucleases were used according to manufacturer’s recommendations (New England Biolabs, Ipswich, MA, USA).

### 4.3. Metabolite Extraction and Analysis

*Streptomyces* strains were grown in 10 mL of TSB for 1 day, and 1 mL of each culture was used to inoculate 50 mL of production medium. Cultures were grown for 7 days at 28 °C. Metabolites were extracted with ethyl acetate from the supernatant, evaporated, and dissolved in methanol. One μL of each sample was separated using a Dionex Ultimate 3000 UPLC (Thermo Fisher Scientific, Waltham, MA, USA), a 10-cm ACQUITY UPLC^®^ BEH C18 column, 1.7 μm (Waters, Milford, MA, USA) and a linear gradient of 0.1% formic acid solution in acetonitrile against 0.1% formic acid solution in water from 5% to 95% in 18 min at a flow rate of 0.6 mL/min. Samples were analyzed using an amaZon speed mass spectrometer or maXis high-resolution LC-QTOF system (Bruker, USA). Data were collected and analyzed with the Bruker Compass Data Analysis software, version 4.1 (Bruker, Billerica, MA, USA). Monoisotopic mass was searched in the natural product database DNP (Dictionary of Natural Products [27]).

### 4.4. Nybomycin Isolation and ^1^H-NMR Spectroscopy

*Streptomyces albus* 4N24 was grown in 30 mL of TSB for 1 day, and 1 mL of the preculture was used to inoculate 100 flasks containing 100 mL of DNPM medium. Cultures were incubated at 28 °C for 7 days. Metabolites from the supernatant were extracted as described above. The crude extract was fractionated by normal phase chromatography on a prepacked silica cartridge (Biotage, Uppsala, Sweden) using hexane, dichloromethane, ethyl acetate, and methanol as the mobile phase. Fractions containing nybomycin were detected by LC-MS analysis. They were pooled together, evaporated, and dissolved in methanol. The sample was further separated by size-exclusion chromatography on an LH 20 Sephadex column (Sigma-Aldrich, USA) using methanol as the solvent. Finally, the sample was separated by semipreparative HPLC (Dionex UltiMate 3000, Thermo Fisher Scientific, USA) using a C18 column (Synergi 10 μm, 250 × 10 mm; Phenomenex, Aschaffenburg, Germany) and a 0.1% formic acid solution in acetonitrile as the mobile phase to obtain nybomycin (0.1 mg). Individual peaks were collected and analyzed by LC-MS as described above. The ^1^H-NMR spectra were recorded on a Bruker Avance 500 spectrometer (Bruker, BioSpin GmbH, Rheinstetten, Germany) at 300 K equipped with a 5 mm BBO probe using deuterated trifluoroacetic acid (Deutero, Kastellaun, Germany) as solvent containing tetramethylsilane (TMS) as a reference. The chemical shifts were reported in parts per million (ppm) relative to TMS. All spectra were recorded with the standard ^1^H pulse program using 128 scans.

### 4.5. Genome Mining and Bioinformatics Analysis

The *S. albus* subsp. *chlorinus* genome was screened for secondary metabolite biosynthetic gene clusters using the antiSMASH [11] online tool (https://antismash.secondarymetabolites.org/#!/start) and the software Geneious 11.0.3 [28]. The DNA sequence of the nybomycin gene cluster was deposited into GenBank under accession number MH924838.

## Figures and Tables

**Table 1:** Proposed functions of genes in nybomycin biosynthetic gene cluster and homology with streptonigrin biosynthetic genes.

Gene	Size (aa)	Proposed Function	GenBank homologue ^1^	Identity/Similarity (%)	Streptonigrin Gene Cluster Homologue ^2^	Identity/Similarity (%)
*orf-07*	268	Streptomycin 3’’-adenylyltransferase	WP_037865927.1	71/77	-	-
*orf-06*	159	Hypothetical protein	WP_027736486.1	86/93	-	-
*orf-05*	163	ATP-binding protein	WP_052413949.1	76/83	-	-
*orf-04*	332	Hypothetical protein	WP_055499466.1	74/82	-	-
*orf-03*	341	Hypothetical protein	WP_030379123.1	71/80	-	-
*orf-02*	494	Hypothetical protein	WP_078869279.1	70/81	-	-
*orf-01*	242	Hypothetical protein	-	-	-	-
*nybA*	475	3-carboxy-cis,cis-muconate cycloisomerase	WP_066029238.1	66/85	*stnL* (AFW04563.1)	71/76
*nybB*	669	FAD-binding protein	WP_066029239.1	77/84	*stnK1* (AFW04562.1)	66/75
*nybC*	325	NADPH:quinone reductase	WP_066029240.1	81/87	*stnH1* (AFW04558.1)	63/71
*nybD*	638	Anthranilate synthase	WP_079145437.1	81/87	*stnM1* (AFW04564.1)	65/75
*nybE*	227	Isochorismatase	WP_066029243.1	84/89	*stnM2* (AFW04565.1)	66/76
*nybF*	402	DAHP synthase	WP_066029245.1	81/87	*stnM3* (AFW04567.1)	62/69
*nybG*	279	Hypothetical protein	-	-	-	-
*nybH*	257	Vicinal oxygen chelate protein	WP_066029246.1	64/72	-	-
*nybI*	222	NAD(P)H:dehydrogenase	WP_066029248.1	90/95	-	-
*nybJ*	135	Hypothetical protein	WP_066029250.1	76/86	-	-
*nybK*	266	*N*-acetyltransferase	WP_066029251.1	80/88	-	-
*nybL*	333	Amidohydrolase	WP_066029254.1	84/90	-	-
*nybM*	354	Acetoacetyl-CoA synthase	WP_066029256.1	82/87	-	-
*nybN*	182	Aromatase/cyclase	WP_066029258.1	78/85	*stnI* (AFW04559.1)	54/67
*nybO*	550	Long-chain acyl-CoA synthetase	WP_066029260.1	85/90	*stnJ* (AFW04560.1)	68/81
*nybP*	476	Salicylate hydroxylase	WP_079145438.0	74/79	*stnH2* (AFW04561.1)	62/72
*nybQ*	376	Hypothetical protein	WP_030685222.1	57/69	-	-
*nybR*	238	NAD-dependent epimerase	WP_066029265.1	82/89	-	-
*nybS*	253	SAM-dependent methyltransferase	WP_079145439.1	89/94	-	-
*nybT*	333	Isopenicillin N synthase family oxygenase	WP_078974705.1	80/89	-	-
*nybU*	342	Isopenicillin N synthase family oxygenase	WP_078974705.1	72/83	-	-
*nybV*	495	MFS transporter	WP_079145411.1	81/87	-	-
*nybW*	243	Transcriptional regulator	WP_079145410.1	82/93	-	-
*nybX*	197	Transcriptional regulator	WP_025356654.1	89/93	-	-
*nybY*	87	Hypothetical protein	WP_086560781.1	68/81	-	-
*nybZ*	219	Transcriptional regulator	WP_057613815.1	79/86	-	-

^1^ NCBI accession numbers are given. ^2^ NCBI accession numbers are shown in parentheses.

## Supplementary Materials

The following are available online at http://www.mdpi.com/1660-3397/16/11/435/s1, Table S1: ^1^H-NMR data for isolated and standard nybomycin; Table S2: Bacterial strains and BACs used in this work; Figure S1: UV chromatograms of crude extracts from *S. albus* 4N24 and *S. albus* Del14; Figure S2: Comparison of LC-MS chromatograms of *Streptomyces albus* 4N24 crude extract and a nybomycin standard; Figure S3: ^1^H-NMR spectra of a nybomycin standard and nybomycin isolated from *S. albus* 4N24; Figure S4: Extracted ion chromatograms of *S. albus* 4M14, *S. albus* 6M11, and *S. albus* 4N24 crude extracts.
