# Supplementary material for: Heterologous Expression of the Nybomycin Gene Cluster from the Marine Strain Streptomyces albus subsp. chlorinus NRRL B-24108
Source: Mar Drugs. 2018 Nov 4;16(11):435. doi: 10.3390/md16110435 (PMC6265801; doi:10.3390/md16110435)
Supplement: Supplementary file 1 [file marinedrugs-16-00435-s001.pdf]

marine drugs

MDPI

Supplementary Information

Identification and Characterisation of the Nybomycin Gene Cluster from the Marine Strain Streptomyces albus subsp. chlorinus NRRL B-24108

Marta Rodríguez Estévez [1], Maksym Myronovskyi [1], Nils Gummerlich [1], Suvd Nadmid [1], Andriy Luzhetskyy [1,2,*]

[1] Pharmazeutische Biotechnologie, Universität des Saarlandes, Saarbrücken 66123, Germany; marta.rodriguezestevez@uni-saarland.de (M.R.E.); m.myronovskyi@googlemail.com (M.M.); nils.gummerlich@uni-saarland.de (N.G.)
[2] Helmholtz-Institut für Pharmazeutische Forschung Saarland, Saarbrücken 66123, Germany
* Correspondence: a.luzhetskyy@mx.uni-saarland.de; Tel.: +49-0681-70223

Tables

Table S1. ^{1}H-NMR (500 MHz, d-TFA) data for isolated and standard nybomycin.

Isolated nybomycin		Standard nybomycin	
Position	δ_H	Position	δ_H
2	6.77 s	2	6.77 s
5	7.09 s	5	7.09 s
6′	2.81 s	6′	2.81 s
7	7.67 s	7	7.67 s
8′	5.52 s	8′	5.52 s
9	8.10 s	9	8.10 s
11′	4.47 s	11′	4.47 s

Table S2. Bacterial strains and BACs used in this work.

Bacterial strain	Features	Reference/Source
Streptomyces albus subsp. *chlorinus* NRRL B-24108	*S. albus* subspecies strain harboring nybomycin biosynthetic gene cluster	[1]
Streptomyces albus Del14	Wild-type strain	[2]
Streptomyces albus 4N24	*S. albus* strain with BAC 4N24 insertion	This work
Streptomyces albus 4M14	*S. albus* strain with BAC 4M14 insertion	This work
Streptomyces albus 6M11	*S. albus* strain with BAC 6M11 insertion	This work
Streptomyces lividans TK24	Wild-type strain	[3]
Streptomyces lividans 4N24	*S. lividans* strain with BAC 4N24 insertion	This work
Escherichia coli ET12567 pUB307	Donor strain for intergeneric conjugation	[4]
Escherichia coli DH10β	General cloning strain	[5]
BACs		
pSMART	AmR; BAC vector	Lucigen (USA)
4N24/4M14/6M11	BACs containing full or partial nybomycin gene cluster	This work

Figures

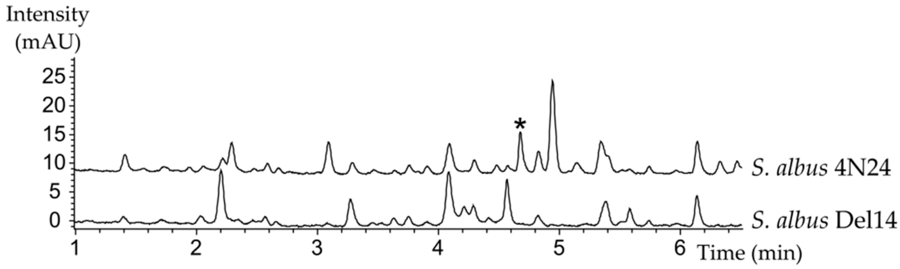

Figure S1. UV chromatograms of crude extracts from *S. albus* 4N24 and *S. albus* Del14. The new peak found in *S. albus* 4N24 crude extract is indicated with an asterisk (*). The profiles correspond to wavelength 285 nm.

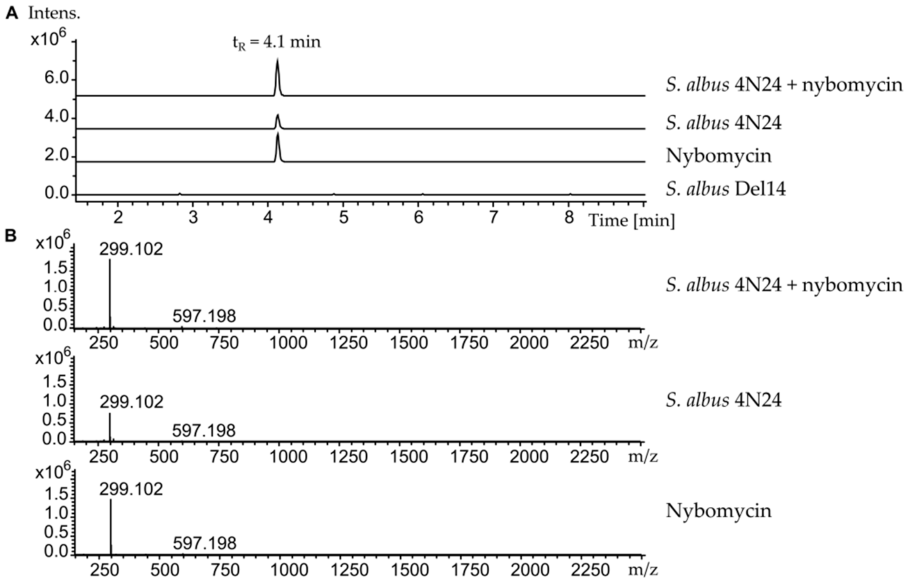

Figure S2. Comparison of LC-MS chromatograms at t_R = 4.1 min of *Streptomyces albus* 4N24 crude extract and a nybomycin standard. (**A**) Extracted ion chromatograms (299.10 ± 0.1 Da) of crude extract from *S. albus* 4N24 supplemented with 0.05 mg/ml of pure nybomycin, crude extract from *S. albus* 4N24 broth culture, a 0.05 mg/ml nybomycin solution in methanol, and crude extract from *S. albus* Del14 broth culture. (**B**) Mass spectra associated to the peak at t_R = 4.1 min from the three upper chromatograms displayed in (A).

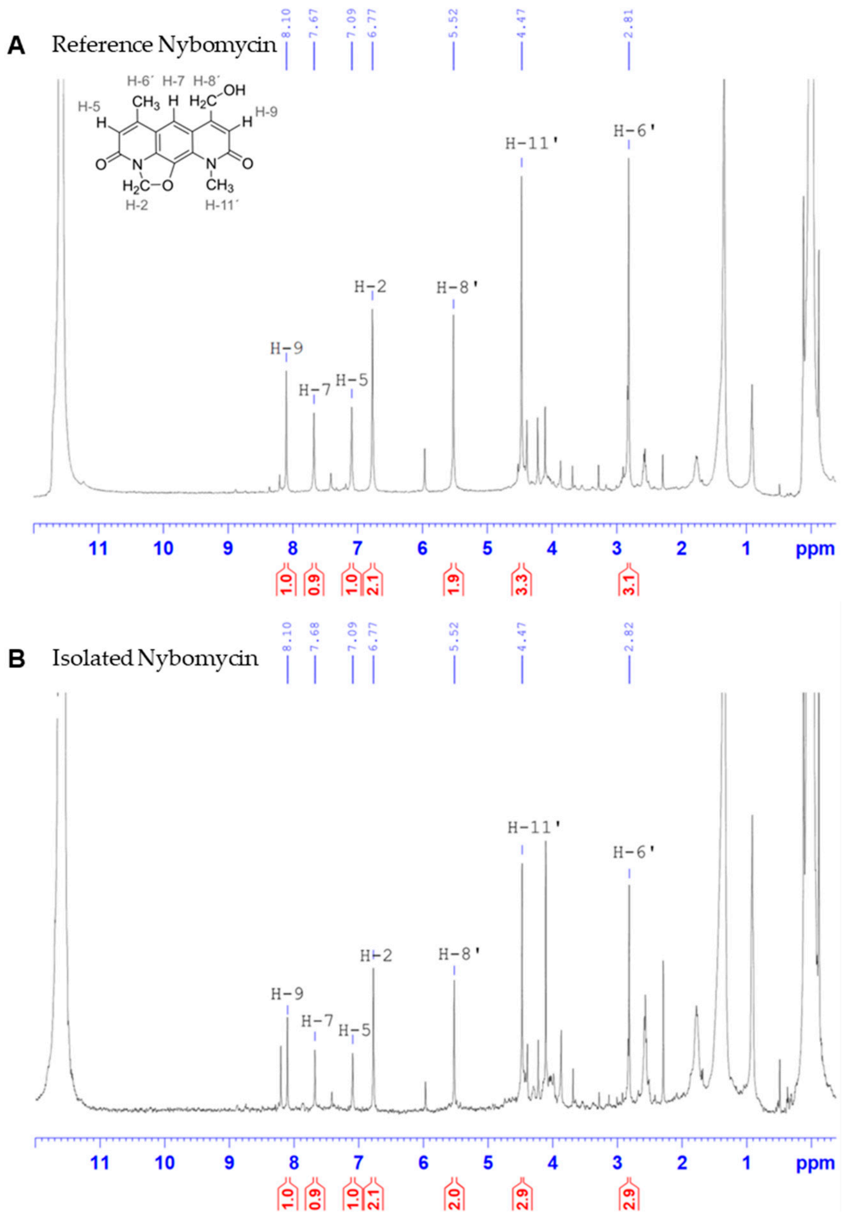

Figure S3. ^{1}H-NMR (500 MHz, d-TFA) spectra of a nybomycin standard and nybomycin isolated from *S. albus* 4N24. ^{1}H-NMR spectra of nybomycin standard (**A**) and the isolated nybomycin (**B**) are identical.

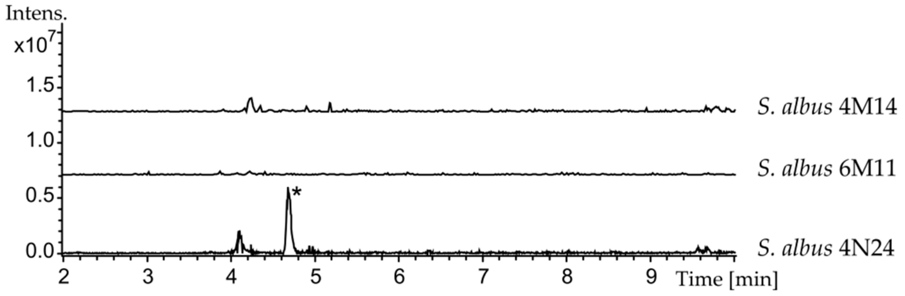

Figure S4. Extracted ion chromatograms of *S. albus* 4M14, *S. albus* 6M11, and *S. albus* 4N24 crude extracts. Nybomycin appears only in *S. albus* 4N24 crude extract, which shows a peak at t_R = 4.7 min (indicated with an asterisk) comprising an $[M+H]^+$ of 299.102 m/z (mass spectrum not shown).

References

1. Hahn, D.R.; Graupner, P.R.; Chapin, E.; Gray, J.; Heim, D.; Gilbert, J.R.; Gerwick, B.C. Albucidin: a novel bleaching herbicide from *Streptomyces albus* subsp. *chlorinus* NRRL B-24108. *J. Antibiot.* **2009**, *62*, 191-194, doi:10.1038/ja.2009.11.
2. Myronovskyi, M.; Rosenkränzer, B.; Nadmid, S.; Pujic, P.; Normand, P.; Luzhetskyy, A. Generation of a cluster-free *Streptomyces albus* chassis strains for improved heterologous expression of secondary metabolite clusters. *Metab. Eng.* **2018**, 10.1016/j.ymben.2018.09.004, doi:10.1016/j.ymben.2018.09.004.
3. Rückert, C.; Albersmeier, A.; Busche, T.; Jaenicke, S.; Winkler, A.; Friethjonsson, O.H.; Hreggviethsson, G.O.; Lambert, C.; Badcock, D.; Bernaerts, K., et al. Complete genome sequence of *Streptomyces lividans* TK24. *J. Biotechnol.* **2015**, *199*, 21-22, doi:10.1016/j.jbiotec.2015.02.004.
4. Flett, F.; Mersinias, V.; Smith, C.P. High efficiency intergeneric conjugal transfer of plasmid DNA from *Escherichia coli* to methyl DNA-restricting streptomycetes. *FEMS Microbiol. Lett.* **1997**, *155*, 223-229.
5. Grant, S.G.; Jessee, J.; Bloom, F.R.; Hanahan, D. Differential plasmid rescue from transgenic mouse DNAs into *Escherichia coli* methylation-restriction mutants. *Proc. Natl. Acad. Sci. USA* **1990**, *87*, 4645-4649.
